# The pattern of healthy diets and zero hunger among households in South-West Nigeria: application of linear approximation to quadratic almost ideal demand system

**DOI:** 10.3389/fpubh.2023.1211479

**Published:** 2023-07-10

**Authors:** Ayodeji Oluwaseun Ogunleke, Seyi Olalekan Olawuyi, Olusegun Jeremiah Ijila

**Affiliations:** ^1^Department of Business, Entrepreneurship and Executive Education, University of Ibadan School of Business, University of Ibadan, Ibadan, Oyo, Nigeria; ^2^Department of Agricultural Economics and Extension, University of Fort Hare, Alice, South Africa

**Keywords:** healthy diets, hunger, households, food, LA-QUAIDS, South-West, Nigeria

## Abstract

Malnutrition and food insecurity remain a significant public health challenge globally, affecting millions of people, particularly in low- and middle-income countries. In Nigeria, the prevalence of malnutrition and food insecurity is high, with a significant burden in the South-West region. Despite several interventions aimed at promoting healthy diets and reducing food insecurity, little is known about the pattern of healthy diets and zero hunger among households, as well as the budget shares on the food items driving these outcomes. Therefore, this study analyzes the pattern of healthy diets and zero hunger among households in South-West Nigeria, using the primary data elicited from 600 rice-consuming households, drawn through a multistage random sampling technique. Descriptive statistics and Linear Approximation to Quadratic Almost Ideal Demand System (LA-QUAIDS) model were used to analyze the dataset. The estimated LA-QUAIDS model was helpful to decipher the relationships that may not be possible to discover using a single-equation model. The finding of this study revealed that rice, especially local (Ofada) rice, is the most consumed food item among the households in the study area with a monthly budget share of 0.195. This is an indication that households spend approximately 19.5% of their monthly food expenditure share on rice consumption. The results also indicated that yam flour (−0.10), cassava-gari (−2.12), and meat (−0.03) are net complements to rice, while the rest of the food items are regarded as net substitutes. The estimated compensated (−1.64) and uncompensated (−1.69) own-price elasticities of local rice, respectively, indicate that local rice variety is price inelastic, and the estimated expenditure elasticity (0.02) suggests that local rice is not a luxury food in the household food basket but a necessity. This study affirms the importance of locally produced foods, especially local rice to the households in the study area. In light of the findings, this study recommends adequate policy aimed at reducing the cost of local food production by boosting domestic production. Apparently, this will technically trigger market price adjustment, as shown in this study, where local rice was estimated to be own-price elastic in nature.

## 1. Introduction

Healthy diets and zero hunger are critical components of sustainable development and poverty reduction targets. According to the United Nations (UN), healthy diets promote overall health and wellbeing and help to prevent chronic diseases, such as obesity, diabetes, and heart disease (1). Similarly, zero hunger is essential for achieving food security, eliminating malnutrition, and eradicating poverty (1). However, despite the global effort to achieve healthy diets and zero hunger, the prevalence of malnutrition and food insecurity remains high in low- and middle-income countries, including Nigeria (2).

From a global perspective, the food system has been weakening over the years owing to multidimensional shocks and other unexpected events, and this situation has exacerbated the systemic issues that are threatening food security and weakening the resilience of the food system (3). The countries' ranking on the Global Food Security Index (GFSI) reveals that the global food environment is seriously deteriorating. Sadly, Nigeria is among the last four weakest performers and bottom-ranked countries with a very low GFSI of 42 points score in the latest ranking. In sub-Saharan Africa, Nigeria is also among the bottom 10 countries whose food security situation is worse (3). This has also contributed to the awful performance of Nigeria in the latest Global Hunger Index (GHI) ranking, which currently stands at a global hunger index score of 27.3 (4).

In recent times, the progress recorded against hunger has largely been stagnated and even worsened in many nations across the world, especially on the prevalence of undernourishment, which reveals a skyrocketing figure of people who do not have regular access to sufficient calories, with as many as 828 million individuals undernourished (4, 5). From a regional perspective, vulnerable populations in sub-Saharan Africa and some other parts of Africa are the most at risk of increased undernourishment because of the persistent unrest and conflicts, as well as other underlying factors in this region, and the proportion of people facing hunger in Congo, Ethiopia, and Nigeria, in particular, is continually increasing (6). Apparently, this threatens the progress toward the ambitious goal of ensuring access to adequate food and healthy diets for all.

In the real sense of it, more than half of the world's population cannot afford a healthy diet (7). In Nigeria, the cost of a healthy diet in Nigeria is approximately $4.1 per person per day, and given this metric, in Nigeria, the percentage of the population who are unable to afford a healthy diet is 95.9% (7). Globally, in approximately 52 countries, where most are residents of Africa, over half of the estimated inhabitants cannot afford a healthy diet (7). While hunger is a global phenomenon, it is particularly acute in African countries. In many countries across sub-Saharan Africa, more than 90% of the population cannot afford a healthy diet. Sub-Saharan Africa is particularly susceptible to extreme climate events and the resulting volatility in food prices is due to the disruption in the supply chain (7–10). This has led to serious inflationary effects in the prices of food items across many African countries, including Nigeria. This evidence suggests that the likelihood of having more people who are unable to afford healthy diets is very high.

Malnutrition and food insecurity are significant public health challenges in Nigeria, and the prevalence of undernutrition is high, with 37% of children younger than 5 years stunted, and 17% wasted (11). Similarly, the prevalence of overweight and obesity is increasing, with 20.8% of adults overweight and 8.5% obese (12). Furthermore, the Food and Agriculture Organization (FAO) estimates that 22.5% of the Nigerian population is food insecure, with more than 90 million people experiencing moderate to severe levels of food insecurity (2). Notable studies have also shown that unhealthy diets and food insecurity are associated with poverty, low education, and poor access to healthy foods (13, 14). The availability and accessibility of healthy foods have been identified as critical determinants of healthy diets and zero hunger (15, 16). However, few notable studies (17–20) have examined the pattern of healthy diets and zero hunger among households in Nigeria.

South-West Nigeria is one of the regions in Nigeria with a high prevalence of malnutrition and food insecurity (2). Malnutrition affects both children and adults, with the highest prevalence among the rural population and low-income households (11). Similarly, food insecurity is prevalent in the region, with many households experiencing inadequate access to food and poor dietary diversity (2). The pattern of healthy diets and zero hunger among households in Africa and, in particular, Nigeria is influenced by several factors. These factors include sociodemographic characteristics, such as income, education, and occupation, as well as environmental and structural factors such as the availability and accessibility of healthy foods, food prices, and food systems (15, 18, 20–22). For instance, households with higher income and education levels are more likely to consume a healthy diet than those with lower income and education levels (21). Similarly, the availability and accessibility of healthy foods in the local food environment are essential for promoting healthy diets and reducing food insecurity (15).

According to Erhabor and Ojogho (23), as well as (24), rice is a staple food crop consumed by nearly every household, but with the recent development of encouraging local production in the nation, Ofada rice represents a good option to focus on owing to its nutritive value which is essential for a healthy diet (24). In Nigeria, efforts to promote healthy diets and zero hunger have been made through various policies and programs. For example, the National Food and Nutrition Policy was launched in 2016 to promote healthy diets and improve nutrition in the country (25). Similarly, the National Social Safety Net Program was established to provide cash transfers to vulnerable households to improve their access to food (26). Meanwhile, the impact of these policies and programs on the pattern of healthy diets and zero hunger among households in South-West Nigeria is largely unfelt, as a majority of the households are finding it difficult to have a healthy diet. Therefore, this study aims to contribute to the existing knowledge by examining the pattern of healthy diets and zero hunger among households in the region, given the consumption expenditures and budget shares by households on various mixtures of food items.

### 1.1. Theoretical and empirical approach

This research utilized the Quadratic Almost Ideal Demand System (QUAIDS) framework to analyze the demand for different food groups and identify patterns of healthy diets among households. The theoretical approach is based on the assumption that households allocate their budget to purchase various food groups to maximize their utility subject to budget constraints. The QUAIDS framework allows for a flexible demand system that accounts for non-linearities and heterogeneity in consumer preferences. The study builds on previous research that has applied the QUAIDS framework to examine food demand in various settings. For instance, Chalak and Kallas (27) used the QUAIDS model to investigate the demand for food in Lebanon and found that consumers are responsive to changes in food prices and income. Similarly, Pascual et al. (28) used the QUAIDS approach to analyze the demand for fruits and vegetables in Spain and found that higher income and education levels are associated with a healthier diet.

Empirically, the model which is an extension of the QUAIDS model is a popular econometric technique used to estimate consumer demand for different goods and services. The QUAIDS model has been widely used in empirical studies of consumer demand, particularly in the analysis of food demand and nutrition. It has been used to estimate the price and income elasticities of demand for various food groups and to analyze the effects of food prices on nutrition outcomes. The LA-QUAIDS model assumes that consumers allocate their budget across different goods and services to maximize utility subject to their budget constraint, and this framework is used to derive a set of demand equations, which explain how changes in prices and income affect the quantities of different goods and services that consumers purchase (29, 30). Importantly, the model provides a flexible and robust framework for estimating consumer demand functions for multiple goods while accounting for heterogeneity in consumer preferences.

According to Pollak (31), the general empirical framework for the LA-QUAIDS model can be expressed as follows:

#### 1.1.1. The demand system

According to Pollak (31), the demand system can be expressed as follows:


$$Q_{ij}=aij+bij^{*}\ln\left(Pj\right)+\gamma j^{*}\ln\left(I\right)+\varepsilon ij,$$

where *Q*_*ij*_ represents the quantity of good *i* that is demanded by the consumer for a given price *Pj* and income *I*.

*aij* represents the constant or intercept term for good *i*.

*bij* is the own-price elasticity of demand for good *i*, which measures how the quantity demanded of good *i* changes in response to a change in its own price.

ln (*Pj*) is the natural logarithm of the price of good *j*.

γ*j* is the income elasticity of demand for good *i*, which measures how the quantity demanded of good *i* changes in response to a change in income.

ln (*I*) is the natural logarithm of the consumer's income.

ε*ij* is the error term representing all other factors that affect the demand for good *i* but are not explicitly included in the model.

#### 1.1.2. The budget constraint

According to Pollak (31), the budget constraint can be expressed as follows:


$$\begin{array}{l} P_{1}Q_{1}+P_{2}Q_{2}+\ldots +P_{n}Q_{n}=I, \end{array}$$

where

*P*_1_, *P*_2_, ..., *P*_*n*_ represent the prices of n different goods and services.

*Q*_1_, *Q*_2_, ..., *Q*_*n*_ represent the quantities of n different goods and services.

*I* represents the consumer's income.

#### 1.1.3. The symmetry restriction

According to Deaton and Muellbauer (29), the symmetry restriction can be expressed as follows:


bij=bji

This assumption implies that the cross-price elasticity of demand between goods *i* and *j* is equal to the cross-price elasticity of demand between goods *j* and *i*.

#### 1.1.4. The Slutsky symmetry restriction

In line with Pollak (31), the Slutsky symmetry restriction can also be expressed as follows:


$$\begin{array}{l} \partial bij/\partial \ln\;\left(Pi\right)=\partial bij/\partial \ln\left(Pj\right) \end{array}$$

This assumption implies that the own-price elasticity of demand for good *i* with respect to the price of good *j* is equal to the own-price elasticity of demand for good *j* with respect to the price of good*i*.

#### 1.1.5. The rank condition

In terms of the rank condition, it requires that the matrix of price and income elasticities is full rank (31), suggesting that it has rank n-1 where n is the number of goods and services in the model.

The LA-QUAIDS model is estimated using data on prices, quantities, and income for different goods and services (31). The estimation process involves solving the demand system equations for the unknown parameters using statistical software. Once the parameters are estimated, they can be used to simulate how changes in prices and income affect the quantities of different goods and services that consumers purchase (31). Overall, the LA-QUAIDS model provides a flexible and useful framework for analyzing consumer demand for different goods and services and can be used to inform policy decisions related to pricing, taxation, and income redistribution.

Overall, the theoretical and empirical approach as applied in this study provides a useful framework for understanding the demand for healthy diets among households. By identifying the patterns of demand for different food items, policymakers can develop targeted interventions to promote healthy eating habits and reduce food insecurity.

## 2. Study area and method of data collection

This study was conducted in the South-West region of Nigeria. The region is one out of six geopolitical zones in the country. The South-West region lies between longitude 2^0^ 31^1^ and 6^0^ 00^1^ East and latitude 6^0^ 21^1^ and 8^0^ 37^1^N and has a total land area of approximately 77,818 km^2^. It shares boundaries in the west, south, east, and north with the Republic of Benin, Gulf of Guinea, Edo and Delta States, and Kwara and Kogi States, respectively. South-West Nigeria's climate is tropical in nature and experiences both rainy and dry seasons. Data were collected with the use of a well-structured questionnaire administered to the representative sample selected for this study. A multistage sampling technique was employed to select the respondents in the study area. The study purposively selected three states, namely, Lagos, Osun, and Ogun because of the predominance of local (Ofada) rice farmers in the areas. The purposive selection was also adopted in the second stage to select two local government areas (LGAs) from each of the selected state. The third stage involved a random selection of two wards, each from the two selected LGAs in each of the states, making it four wards from each selected state based on a lucky-dip approach (24, 32). The final stage used a random proportionate-to-size sampling technique to select 600 respondents (households) from all the chosen LGAs. This was applied because of the population variation that exists across the villages in the selected LGAs. Hence, this represents the representative sample used for the research.

This study collected information on the following commonly consumed food items: rice (Main staples), Maize (Main staples), Yam tuber (Main staples), Yam flour Main staples, Beans (Pulses), Cassava-Gari (Main staples), Plantain (Fruit), Vegetable (Vegetables), Fruits (Fruit), Meat (Meat and fish), Beverages (Condiments), Fats and Oil (Oil). According to World Food Programme (33), the food items are broadly grouped into different food groups, as shown in parentheses beside the food items. This also requires further analysis of attaching weights and measurements if the study is interested in the food consumption score of the sampled households, which is outside the scope of this study. Hence, the main focus of this study was on the budget share analysis of these food items and to mirror the households' pattern of consumption expenditure on the food items using budget share analysis.

## 3. Data analysis approach and econometrics modeling

### 3.1. Linear approximated quadratic almost ideal demand system (LA-QUAIDS)

The discourse on the approach to the LA-QUAIDS model was adapted from the study by Diewert (34), Moschini (35), and Matsuda (36). At the stage of linearization estimation of the QUAIDS, “the translog price aggregator term, i.e., *f(p)* and the Cobb–Douglas price aggregator term, i.e., g(p) will be replaced with a composite variable which does not depend on unknown parameters from the general QUAIDS model (36)”.

Stone's (geometric) price index ln *h*(p^*^) represents the widely used composite variable for the approximation of f(p) (29), which is expressed as follows:


(1) $$\begin{array}{l} \ln\;h\left(p^{*}\right)=\Sigma_{i=1}^{n}w_{i}\ln\;p_{i}. \end{array}$$

Moreover, the price index was usually applied in the linearization of the Cobb–Douglas price aggregator part of the model (34, 36). This is also expressed as follows:


(2) $$\begin{array}{l} \ln\;p^{z}=\Sigma_{i=1}^{n}\left({\bar{w}}_{it}-{\bar{w}}_{it}^{0}\right)\left(\ln\;p_{t}-\ln\;p_{it}^{0}\right). \end{array}$$

The budget shares and prices, denoted by the superscript zero, are the associated base budget share and base price value. Meanwhile, Diewert (34) suggested the introduction of the “chained principle” which has to do with incorporating the time values (*t*−1) as the base for time *t*. With this, equation (4) can be transformed to read:


(3) $$\begin{array}{l} \ln\;p^{z}=\Sigma_{i=1}^{n}\left({\bar{w}}_{it}\right)\left(\ln\;p_{t}^{}\right). \end{array}$$

Given the processes above, one can derive equation (14), showing the linear approximated version of the QUAIDS model, which is the LA-QUAIDS, expressed in the following form:


(4) $$\begin{array}{lll} w_{t} & = & \alpha_{i}+\overset{n}{\underset{j=1}{\sum }}Y_{ij}\ln\;p_{j}+\beta_{it}\left[\ln\;m-\overset{n}{\underset{i=1}{\sum }}w_{it}^{}\ln\;p_{it}\right]\\ +\frac{\lambda_{i}}{\overset{n}{\underset{i=1}{\sum }}\left({\bar{w}}_{it}\right)\left(\ln\;p_{it}\right)}{\left[\ln\;m-\overset{n}{\underset{i=1}{\sum }}w_{it}\ln\;p_{it}\right]}^{2} \end{array}$$

Differentiating equation (4) with respect to income and price results in the following:


(5) $$\frac{\partial w_{i}}{\partial \ln m}\equiv \mu_{i}=\beta_{i}+\frac{2{\text{λ}}_{i}}{\sum_{i=1}^{n}\left({\bar{w}}_{it})(\ln p_{it}\right)}(\ln m-\sum_{i=1}^{n}\left(w_{it})(\ln p_{it}\right)$$

(6) $$\begin{array}{l} \;\frac{\partial w_{i}}{\partial \ln p_{j}}\equiv \mu_{ij}=\gamma_{ij}-\mu_{i}w_{jt}\;\\ \;-\frac{{\text{λ}}_{i}\left({\bar{w}}_{jt}\right)}{\Sigma_{i=1}^{n}\left({\bar{w}}_{it}\right)\left(\ln p_{it}\right)}(\ln m-\Sigma_{i=1}^{n}\left(w_{it}\right){\left(\ln p_{it}\right)}^{2} \end{array}$$

Expenditure elasticity *e*_*i*_ is calculated as follows:


(7) $$\begin{array}{l} e_{i}=\frac{\mu_{i}}{w_{i}}+1. \end{array}$$

Uncompensated price elasticities $e_{ij}^{u}$ are calculated as follows:


(8) $$\begin{array}{l} e_{ij}^{u}=\frac{\mu_{ij}}{w_{i}}-\partial_{ij}, \end{array}$$

where ∂_*ij*_ is the Kronecker delta (∂_*ij*_ = 1 if i = j and ∂_*ij*_ = 0 if *i*≠*j*). Recovery of the compensated price elasticities $e_{ij}^{c}$ was made using the Slutsky derivative expressed in elasticity form as follows:


(9) $$\begin{array}{l} e_{ij}^{c}=e_{ij}^{u}+e_{i}w_{j}. \end{array}$$

Expression in equation (10) was used to recover symmetric values of compensated cross-price elasticity estimates:


(10) $$\begin{array}{l} e_{ij}^{c}=\left(\frac{w_{j}}{w_{i}}\right)e_{ij}^{c}+w_{j}\left(e_{j}-e_{i}\right), \end{array}$$

where w's are budget shares of *i*th and *j*th good, and *e*_*j*_.*e*_*j*_ are expenditure elasticities of *j*th and *i*th goods, respectively.

The pattern of consumption expenditure and the inter-relationships among the 13 food items were captured using the LA-QUAIDS model. In the analyses, expenditure, own-price, and cross-price demand elasticities (both uncompensated and compensated) were all estimated for the food items considered in the study.

## 4. Results and discussion

The results of expenditure, own-price, and cross-price demand elasticities (both uncompensated and compensated) are presented sequentially.

The result in Table 1 revealed that the households' budget shares on local rice in Lagos, Ogun, and Osun States were 0.11, 0.14, and 0.09, respectively. This implies that 11%, 14%, and 9% of the households' food expenditure were spent on local rice consumption, and from a general perspective of rice consumption (both local and foreign), approximately 19.5%, 23%, and 17.5% of the budgets were expended for the period under consideration. The households' budget share on local rice for the aggregated households of the study is 0.11, which indicates that households spent 11% of their food expenditure on local rice variety and 19.5% of food expenses on both local and foreign rice consumption. However, 19.5% of households' expenditure on both local and foreign rice was found to be below that of the North-West geopolitical zone, and the nation's average was reported to be 28.39% and 30.63%, respectively (37). The highest budget share is associated with rice consumption (19.36%), and the lowest budget share is associated with plantain (1.91%). The budget share of fat and oil stands at the second highest. Households' total expenditure for all of the food items considered for the study is N=79,651,455 ($218,223) for the period under review. This finding lends credence to the growing trend in households' rice consumption preference over other food items and therefore, the central position of rice in households' food basket. The higher proportion of households' expenditure on rice, both local and foreign varieties, agrees with Omonona et al.'s study (38) and Erhabor and Ojogho (23) also established that rice had the highest proportion of households' monthly food expenditure relative to other food items in their study. The implication is that sudden changes in households' food expenditure would affect the households' rice consumption.

**Table 1:** Summary statistics: food expenditure and budget share on food items.

	**Food expenditure (N**=**)**				**Budget share (%)**			
**Food item**	**Lagos**	**Ogun**	**Osun**	**Pooled**	**Lagos**	**Ogun**	**Osun**	**Pooled**
Local Rice	2942650	3077400	2649325	8669375	11.22	13.86	8.75	11.01
Foreign Rice	2160500	1946000	2457500	6564000	8.24	8.76	8.11	8.34
Rice	5103150	5023400	5290950	15417500	19.46	22.62	17.47	19.59
Maize	585000	583800	559050	1727850	2.23	2.63	1.85	2.20
Yam	998500	695250	1392950	3086700	3.81	3.13	4.60	3.92
Yam Flour	1397500	632400	1907100	3937000	5.33	2.85	6.30	5.00
Beans	1091690	635950	1554050	3281690	4.16	2.86	5.13	4.17
Cassava (Gari)	869200	540720	1270350	2680270	3.32	2.44	4.19	3.41
Plantain	536700	457200	528600	1522500	2.05	2.06	1.75	1.93
Vegetable	2246300	2433240	1993600	6673140	8.57	10.96	6.58	8.48
Fruits	1142230	182880	2226250	3551360	4.36	0.82	7.35	4.51
Meat	2514930	1707480	3452850	7675260	9.59	7.69	11.40	9.75
Beverages	1661800	1529550	1816450	5007800	6.34	6.89	6.00	6.36
Fat & Oil	2967150	2760600	3189250	8917000	11.32	12.43	10.53	11.33
**Total food expenditure**	**26217300**	**22205870**	**30288275**	**78711445**				

Expenditure figures in Nigeria Naira (1$ = N=365) at the time of data collection, Budget share figures in percentages.

Field survey, 2019.

Given the observation on the budget share attached to the prevalent food items consumed by households in the study area, one can infer that a large proportion of the budget was expended on the main staple food items, suggesting that there was no significant diversity in the food item matrix of the household. Evidently, this can have implications on the health outcomes of the household members and ultimately lead to a serious healthy diet crisis.

### 4.1. LA-QUAIDS elasticity estimates

Given the estimates of the parameters, this study calculated the expenditure, uncompensated and compensated own-price, and the cross-price elasticities for local rice and other food items consumed by households. It is important to reiterate that the elasticity expression shown in equations (18, 19) was applied. The “*uncompensated cross-price elasticities indicate the gross substitution and gross complementary effects while its compensated counterpart distinguishes between net substitutes and net complements*”. In addition, the expenditure elasticity shows the percentile increase or decrease (change) in the consumption of a food item as a result of a percentile increase or decrease in the expenditure of the other food items included in the demand equation.

Tables 2, 3 show the calculated uncompensated and compensated own price, cross-price, and expenditure elasticities for each of the food items. The expenditure elasticity of rice as presented in Table 2 indicates that local rice is a normal good as shown by the positive sign of its expenditure elasticity and is expenditure inelastic as its expenditure elasticity lies between one and zero. This suggests that local rice is not a luxury in the households' food basket but a necessity, and a unit increase in the households' income would be proportionately less than the increasing demand for local rice by a magnitude of 0.02. This finding agrees with Onyeneke et al. (39) and Gyimah-Brempong and Kuku-Shittu (40) that local rice is a necessity and not luxury food but disagrees with Omonona et al. (38) who posited that rice is an inferior good, as indicated by the negative sign of its expenditure elasticity (−5.2837), and this is an expenditure elastic food item. This could be because what was obtainable in terms of the nation's economy at the time of their research is quite different from the present economic reality. In Nigeria, rice is perhaps the most consumed food item, and this can be established based on the amount spent ($1.0 billion) yearly on the import of this food item (32). The status of local rice, being a necessity in the households' food basket, is a pointer to the growing consumer preference in Nigeria. The calculated expenditure elasticities also revealed that all the food items were expenditure inelastic, meaning that the food items are not luxury goods but necessities.

**Table 2:** Expenditure elasticities and uncompensated own-price and cross-price demand elasticities estimated through LA-QUAIDS.

**Variable**	**WO**	**WE**	**WM**	**WY**	**WYF**	**WB**	**WG**	**WP**	**WV**	**WF**	**WMT**	**WBV**	**WFO**	**Ex p**
WO	**−1.69**	−0.04	**0.01**	**0.02**	**0.02**	0.01	**0.02**	**0.02**	**0.01**	**0.02**	0.01	**0.01**	0.01	**0.02**
	0.00	0.34	0.02	0.01	0.02	0.18	0.01	0.00	0.02	0.00	0.13	0.02	0.47	0.00
WE	0.00	**−1.33**	0.00	0.00	0.00	0.00	0.00	0.00	0.00	0.00	0.00	0.00	0.00	**0.31**
	0.95	0.00	0.25	0.18	0.45	0.08	0.36	0.74	0.44	0.99	0.93	0.73	0.78	0.00
WM	0.18	0.84	**−8.15**	**−0.85**	**2.28**	**0.75**	**1.25**	0.02	**−0.18**	−0.07	**1.37**	**1.79**	**0.99**	**0.01**
	0.53	0.08	0.00	0.00	0.00	0.00	0.00	0.68	0.01	0.43	0.00	0.00	0.00	0.00
WY	0.00	**−0.21**	**−0.05**	**−0.53**	**−0.02**	**−0.02**	**−0.03**	**−0.02**	**−0.02**	**−0.02**	**−0.03**	0.00	**−0.02**	**−0.04**
	0.83	0.00	0.00	0.00	0.00	0.00	0.00	0.00	0.00	0.00	0.00	0.20	0.00	0.00
WYF	−0.10	−0.64	**5.60**	**0.58**	**−1.69**	**−0.48**	**−0.76**	0.02	**0.11**	0.08	**−0.98**	**−1.33**	**−0.69**	**0.01**
	0.59	0.05	0.00	0.00	0.00	0.00	0.00	0.66	0.01	0.18	0.00	0.00	0.00	0.00
WB	3.33	12.59	**−125.46**	**−12.95**	**25.18**	**10.72**	**18.03**	**3.93**	−1.64	−0.20	**21.51**	**25.52**	**13.51**	**0.03**
	0.44	0.08	0.00	0.00	0.00	0.00	0.00	0.00	0.09	0.88	0.00	0.00	0.00	0.00
WG	−2.12	−9.24	**89.94**	**9.14**	**−17.16**	**−8.20**	**−13.38**	**−3.26**	1.07	0.24	**−15.86**	**−18.60**	**−9.90**	**0.01**
	0.49	0.08	0.00	0.00	0.00	0.00	0.00	0.00	0.12	0.81	0.00	0.00	0.00	0.00
WP	0.33	1.76	**−16.90**	**−1.72**	**3.85**	**1.50**	**2.38**	**0.44**	**−0.26**	−0.12	**2.96**	**3.57**	**1.94**	0.00
	0.56	0.07	0.00	0.00	0.00	0.00	0.00	0.00	0.04	0.49	0.00	0.00	0.00	0.56
WV	0.87	1.65	**−19.97**	**−2.31**	**3.03**	**1.83**	**3.03**	**1.15**	**−1.00**	0.23	**3.80**	**3.71**	**2.03**	**−0.03**
	0.21	0.17	0.00	0.00	0.00	0.00	0.00	0.00	0.00	0.32	0.00	0.00	0.00	0.00
WF	1.06	5.45	**−55.36**	**−5.20**	**9.58**	**4.97**	**7.71**	**0.91**	−0.66	−0.95	**9.91**	**11.14**	**6.11**	0.00
	0.57	0.09	0.00	0.00	0.00	0.00	0.00	0.01	0.11	0.10	0.00	0.00	0.00	0.09
WMT	−0.03	−0.52	**6.68**	0.27	**−1.67**	**−0.56**	**−1.36**	**0.14**	**0.23**	−0.02	**−2.83**	**−1.53**	**−0.81**	**0.05**
	0.91	0.16	0.00	0.11	0.00	0.00	0.00	0.00	0.00	0.68	0.00	0.00	0.00	0.00
WBV	0.04	**0.66**	**−1.24**	**−0.09**	**0.16**	**0.10**	**0.31**	−0.01	**−0.08**	−0.01	**0.13**	**0.29**	**0.47**	**−0.05**
	0.30	0.00	0.00	0.00	0.00	0.00	0.00	0.46	0.00	0.24	0.00	0.00	0.00	0.00
WFO	0.53	2.88	**−31.18**	**−2.99**	**7.81**	**2.70**	**4.74**	**−0.66**	**−0.85**	−0.37	**5.15**	**6.29**	**2.84**	**−0.01**
	0.61	0.10	0.00	0.00	0.00	0.00	0.00	0.00	0.00	0.24	0.00	0.00	0.00	0.04

Numbers just below estimated coefficients represent *p*-values. Estimated coefficients in bold font show the parameters that are statistically significant at alpha level 0.10. Abbreviations of food items are as follows: WO, local rice; WE, Foreign rice; WM, Maize; WY, Yam; WFY, Yam Flour; WB, Beans; WG, Cassava (Gari); WP, Plantain; WV, Vegetable; WF, Fruits; WMT, Meat; WBV, Beverages; and WFO, Fat & Oil. Exp, Expenditure elasticity.

**Table 3:** Compensated own-price and cross-price demand elasticities estimated through LA-QUAIDS.

**Variable**	**WO**	**WE**	**WM**	**WY**	**WYF**	**WB**	**WG**	**WP**	**WV**	**WF**	**WMT**	**WBV**	**WFO**
WO	**−1.64**	−0.03	**0.02**	0.01	0.01	0.00	0.01	**0.02**	**0.02**	**0.02**	0.01	**0.01**	0.01
	0.00	0.45	0.01	0.38	0.06	0.41	0.09	0.00	0.00	0.00	0.39	0.03	0.43
WE	0.01	**−1.51**	0.00	0.01	0.00	0.00	0.00	0.00	0.00	0.00	0.00	0.00	0.00
	0.16	0.00	0.33	0.10	0.43	0.59	0.61	0.78	0.29	0.93	0.87	0.62	0.84
WM	0.15	**1.59**	**−8.74**	**1.09**	**2.08**	**0.74**	**1.16**	0.02	**−0.48**	−0.08	**2.40**	**1.73**	**1.00**
	0.61	0.00	0.00	0.00	0.00	0.00	0.00	0.68	0.00	0.38	0.00	0.00	0.00
WY	0.01	**−0.23**	**−0.05**	**−0.52**	**−0.03**	**−0.02**	**−0.03**	**−0.02**	**−0.03**	**−0.02**	**−0.03**	0.00	–**0.02**
	0.51	0.00	0.00	0.00	0.00	0.00	0.00	0.00	0.00	0.00	0.00	0.16	0.00
WYF	−0.08	**−1.10**	**5.99**	**−0.71**	**−1.53**	**−0.48**	**−0.70**	0.02	**0.31**	0.08	**−1.69**	**−1.28**	**−0.70**
	0.67	0.00	0.00	0.00	0.00	0.00	0.00	0.65	0.00	0.16	0.00	0.00	0.00
WB	2.76	**24.66**	**−134.18**	**15.59**	**22.63**	**10.51**	**16.68**	**3.93**	**−5.88**	−0.34	**37.33**	**24.72**	**13.71**
	0.53	0.00	0.00	0.00	0.00	0.00	0.00	0.00	0.00	0.80	0.00	0.00	0.00
WG	−1.76	**−17.57**	**96.24**	**−11.72**	**−15.42**	**−8.02**	**−12.43**	**−3.26**	**4.03**	0.35	**−27.31**	**−18.07**	**−10.05**
	0.58	0.00	0.00	0.00	0.00	0.00	0.00	0.00	0.00	0.72	0.00	0.00	0.00
WP	0.27	**3.24**	**−18.07**	**2.14**	**3.49**	**1.47**	**2.20**	**0.44**	**−0.82**	−0.14	**5.08**	**3.45**	**1.98**
	0.65	0.00	0.00	0.00	0.00	0.00	0.00	0.00	0.00	0.43	0.00	0.00	0.00
WV	0.79	**4.11**	**−21.36**	**2.32**	**2.60**	**1.76**	**2.79**	**1.16**	**−1.69**	0.22	**6.44**	**3.69**	**2.06**
	0.27	0.00	0.00	0.00	0.00	0.00	0.00	0.00	0.00	0.35	0.00	0.00	0.00
WF	0.85	**10.44**	**−59.22**	**7.93**	**8.55**	**4.82**	**7.17**	**0.90**	**−2.41**	−1.04	**16.90**	**10.84**	**6.21**
	0.66	0.00	0.00	0.00	0.00	0.00	0.00	0.01	0.00	0.07	0.00	0.00	0.00
WMT	−0.02	**−0.92**	**7.14**	**−1.74**	**−1.52**	**−0.50**	**−1.30**	**0.14**	**0.47**	−0.02	**−3.60**	**−1.47**	**−0.83**
	0.94	0.02	0.00	0.00	0.00	0.00	0.00	0.00	0.00	0.70	0.00	0.00	0.00
WBV	0.02	**0.14**	**−1.34**	**0.21**	**0.13**	**0.09**	**0.29**	0.00	**−0.14**	−0.01	**0.23**	**0.31**	**0.47**
	0.63	0.04	0.00	0.00	0.00	0.00	0.00	0.54	0.00	0.25	0.00	0.00	0.00
WFO	0.38	**5.49**	**−33.33**	**4.17**	**7.07**	**2.63**	**4.41**	**−0.66**	**−1.93**	−0.40	**8.86**	**6.03**	**2.96**
	0.73	0.00	0.00	0.00	0.00	0.00	0.00	0.00	0.00	0.20	0.00	0.00	0.00

Numbers just below estimated coefficients represent *p*-values. Estimated coefficients in bold font show the parameters that are statistically significant at alpha level ≤ 0.10. Abbreviations of food items are as follows: WO, local rice; WE, Foreign rice; WM, Maize; WY, Yam; WFY, Yam Flour; WB, Beans; WG, Cassava (Gari); WP, Plantain; WV, Vegetable; WF, Fruits; WMT, Meat; WBV, Beverages; and WFO, Fat & Oil.

Uncompensated and compensated own-price elasticities of demand for 9 (9) out of 13 food items are negative and are statistically significant. The values of compensated and uncompensated own-price elasticities for local rice are −1.64 and −1.69, respectively, which is an indication that the food is price-sensitive (41). Here, in line with the demand theory, consumers are likely to respond to any changes in the price. The absolute value of the own-price elasticity of local rice is higher than the absolute values of its cross-price elasticity. This implies that the demand for local rice is more responsive to its price than the prices of other food items. This result is in line with the findings of Makama et al. (42) and Kuku-Shittu and Pradesha (43) that local rice uncompensated and compensated own-price elasticities are negative for rural and urban consumers in Nigeria. The estimated compensated own-price and cross-price elasticities of local rice, as shown in Table 3, are lower than its uncompensated own-price and cross-price elasticities. This implies that the substitution effect surpasses the income effect. This finding supports Erhabor and Ojogho (23) who found that the uncompensated elasticities of rice were higher than the compensated elasticities of rice. On the other hand, the compensated own-price elasticity of local rice was similar to the uncompensated own-price elasticity in being price elastic and negative. This suggests that a unit increase in the price of local rice would be more than a proportionate decrease in demand for local rice by a magnitude of 1.64. This agrees with the findings of Hoang (44) and Obalola et al. (45) who noted that the demand for rice with respect to price is relatively elastic compared to other foods.

However, Gari is the most price-sensitive out of all the food items, having a compensated own-price elasticity of demand of −12.43. Even though there is a small budget share associated with Gari (~3%) compared to other food items, it is the most expensive out of the 13 food items considered in this study. Given this high price of Gari, consumers may be more sensitive to changes in its price. The compensated own–price elasticities of demand for foreign rice, maize, yam flour, beans, vegetable, fruits, meat, and fat and oil are −1.51, −8.74, −1.53, 10.51, −1.69, −1.04, −3.60, and 2.97, respectively, indicating elastic demands. Yam has the most inelastic compensated own–price elasticity of demand, which is −0.06. This finding is similar to the study by Gyimah–Brempong and Kuku–Shittu (40) that most food items are complements for rice consumption among households in Nigeria. In addition, Demont et al. (46) who carried out research on rice demand in Senegal found that local rice and imported rice are characterized by low substitutability and that compensated cross–price elasticity estimates between rice and other food commodities are small and mostly insignificant.

Moreover, 3 out of 13 compensated cross–price elasticities have negative signs with local rice (Yam flour = −0.10, Gari = −2.12, and meat = −0.03), indicating net complements to local rice consumption. Gari has the highest complimentary value with local rice, followed by yam flour, then meat, while the remaining 10 food items compensated cross-price elasticities are indicative of net substitutes. In total, 66 out of 156 (42%) compensated cross-price elasticities have negative signs indicating net complements, and 58% of compensated cross-price elasticities are indicative of net substitutes.

### 4.2. Limitations of the study and areas for further research

This research was limited to the analysis of the households' budget share on the food items commonly consumed in the study area. While we acknowledged that each of the food items identified in the study area also belongs to one of the food groups or the other, as highlighted in WFP (2008), further study and analysis can be carried out in this aspect by conducting an in-depth food security analysis using the recommended weights and measurements on the food items, to estimate the households' food consumption score.

## 5. Contribution of the study

This study underscores the importance of having access to good-quality and healthy foods by people to cater to their dietary needs, given various mixes of food items required for adequate human functionality in society. The research probed on the consumption-expenditure pattern and households' budget shares on various food items commonly consumed by people through the estimation of households' price and demand elasticity which only a few studies have dealt with in the past using other study locations, different from this study area. Besides, most of this study used the QUAIDS model, while this study applied an extended version of the model known as the LA-QUAIDS model. This is very important to guide the policy maker in the design and implementation of necessary policy actions that can encourage domestic production, increase food distribution and supply, and ultimately reduce the market prices of locally produced food items; all aiming at self-sufficiency in local food production.

## 6. Conclusion

This study used a linear approximated QUAIDS model to analyze households' demand for local rice in the South-West region of Nigeria. The findings from this study reveal that households in Nigeria consume local rice to a reasonable extent and that local rice does not represent a luxury good based on the calculated expenditure elasticities because they are all expenditure inelastic. Uncompensated and compensated own-price elasticities of demand for 9 (9) out of 13 food items are negative, which is consistent with demand theory, and they are statistically significant. The value of compensated and uncompensated own-price elasticities for local rice was found to be −1.64 and −1.69, respectively, which suggests that local rice is price-sensitive. That is, consumers are likely to be affected by any changes in the price of local rice. In light of the findings of this study, it is recommended that adequate policy aimed at boosting local rice production and increasing its supply should be pursued. This could invariably connote a price-reduction strategy, by default. Suffice to say that increased supply will force down the price of local rice because the food crop was shown to be own-price elastic.

## Data availability statement

The raw data supporting the conclusions of this article will be made available by the authors, without undue reservation.

## Ethics statement

The studies involving human participants were reviewed and approved by University Research Ethics Committee. The patients/participants provided their written informed consent to participate in this study.

## Author contributions

All authors listed have made a substantial, direct, and intellectual contribution to the work and approved it for publication.
